# Aberrant individual structure covariance network in patients with mesial temporal lobe epilepsy

**DOI:** 10.3389/fnins.2024.1381385

**Published:** 2024-05-09

**Authors:** Yuda Huang, Ningrui Wang, Wei Li, Tao Feng, Huaqiang Zhang, Xiaotong Fan, Sichang Chen, Yihe Wang, Yongzhi Shan, Penghu Wei, Guoguang Zhao

**Affiliations:** ^1^Department of Neurosurgery, Xuanwu Hospital Capital Medical University, Beijing, China; ^2^Beijing Municipal Geriatric Medical Research Center, Beijing, China; ^3^School of Public Health and Management, Ningxia Medical University, Yinchuan, China; ^4^Clinical Research Center for Epilepsy Capital Medical University, Beijing, China

**Keywords:** mesial temporal lobe epilepsy, hippocampal sclerosis, structure covariance network, gray matter volume, drug resistant epilepsy

## Abstract

**Objective:**

Mesial temporal lobe epilepsy (mTLE) is a complex neurological disorder that has been recognized as a widespread global network disorder. The group-level structural covariance network (SCN) could reveal the structural connectivity disruption of the mTLE but could not reflect the heterogeneity at the individual level.

**Methods:**

This study adopted a recently proposed individual structural covariance network (IDSCN) method to clarify the alternated structural covariance connection mode in mTLE and to associate IDSCN features with the clinical manifestations and regional brain atrophy.

**Results:**

We found significant IDSCN abnormalities in the ipsilesional hippocampus, ipsilesional precentral gyrus, bilateral caudate, and putamen in mTLE patients than in healthy controls. Moreover, the IDSCNs of these areas were positively correlated with the gray matter atrophy rate. Finally, we identified several connectivities with weak associations with disease duration, frequency, and surgery outcome.

**Significance:**

Our research highlights the role of hippo-thalamic-basal-cortical circuits in the pathophysiologic process of disrupted whole-brain morphological covariance networks in mTLE, and builds a bridge between brain-wide covariance network changes and regional brain atrophy.

## 1 Introduction

Mesial temporal lobe epilepsy (mTLE) is a complex neurological disorder characterized by diverse seizure symptoms with strong individual heterogeneity. An increasing number of studies have classified mTLE as a widespread global network disorder that extends beyond the epileptogenic zone despite being traditionally regarded as focal epilepsy (Spencer, 2002; Berg et al., 2010; Englot et al., 2020). Network theories provide a more comprehensive explanation of epilepsy mechanisms and potentially contribute new treatment measures in the future (Wei et al., 2022). The structural covariance network (SCN) derived from structural magnetic resonance imaging (MRI) depicts the coordinated variation in the morphological properties of brain regions (Mechelli et al., 2005). The SCN provides a promising method of revealing alterations in human brain structure, especially in developmental relationships between different brain regions, which obeys the basic organizational principles for the anatomical network in the human brain (He et al., 2007; Zielinski et al., 2010). Structural covariance was related to white matter fiber connections and functional network organization between separate brain regions (Gong et al., 2012; Alexander-Bloch et al., 2013). In mTLE, abnormalities in the covariance patterns between the mesial temporal lobe and extensive cortical regions have been demonstrated in earlier studies (Bernhardt et al., 2008; Mueller et al., 2009). mTLE patients exhibit reconfigurations of structural covariance network topology, involving increases in path length and clustering coefficient, and the alterations intensify over the seizure duration (Bernhardt et al., 2011). Moreover, structural covariance network patterns reflect a potential association with postoperative seizure outcomes in mTLE patients (Bernhardt et al., 2011). Molding structural covariance at the group level can greatly contribute to explaining pathophysiological mechanisms and improving clinical diagnosis and treatment in mTLE patients.

There is broad consensus that mTLE is a highly heterogeneous neurological disorder in terms of pathophysiology, clinical manifestations, and neuroimaging features (Paradiso et al., 1995; Coan and Cendes, 2013; Thijs et al., 2019). Surgery for mTLE is supposed to be an efficient method for controlling seizure onset, but only 76% of patients reach a “seizure-free” outcome postoperatively (Wiebe et al., 2001). However, traditional studies on structural covariance networks limited to the group level cannot sufficiently identify individual differences in heterogeneous diseases such as epilepsy and are unable to describe the correspondence between individual network patterns and clinical characteristics. It is necessary to study the morphological similarity alterations between brain regions of mTLE at the individual level.

Recently, the method of constructing an individual specialized SCN was introduced to depict subject-specific covariance variability in healthy people (Zhao et al., 2021) and to outline covariance alterations in neuropsychiatric disease patients (Liu et al., 2021; Shen et al., 2021; Han et al., 2022). The individualized SCN is measured according to the correlation of multiple morphometric features (Li et al., 2017; Seidlitz et al., 2018; Morgan et al., 2019; Zhao et al., 2021), regional probability density distribution (Kong et al., 2014; Wang et al., 2016) or network perturbation approaches (Kim et al., 2016; Wolfers et al., 2018). Liu et al. (2021) recently proposed a promising approach for mapping individual structural covariance networks (IDSCNs) based on a sample-specific group network perturbation method (Liu et al., 2016). They found that individual structural covariance alterations in the left hippocampus–bilateral putamen/globus pallidus were related to affective symptoms in schizophrenia (Liu et al., 2021). Han et al. correspondingly demonstrated heterogeneity in depression and elucidated the relationship between the subcortical-cerebellum network and the pathophysiological mechanism of depression (Han et al., 2022). Currently, few studies are focusing on the SCN changes of mTLE at the individual level. An attempt has been made by Drenthen et al. to describe the structural covariance network characteristics of focal epilepsy at the individual level (Drenthen et al., 2018). However, the insufficient sample size and heterogeneity distribution of the epileptogenic focus limit the exploration of the morphological covariance features of mTLE. Constructing the IDSCN of mTLE patients would greatly assist in uncovering individual morphological covariance, depicting node-level anomalies through differentiated networks and revealing their relevance to clinical features. However, there are gaps in applying the IDSCN method in mTLE. Moreover, mTLE patients have extensive brain structural alterations, while the relationship between focal brain atrophy and global covariation changes remains unclear.

To solve the abovementioned issues, we studied a large cohort of 71 patients with a single clinical diagnosis of mesial temporal lobe epilepsy with hippocampal sclerosis. This study adopted the IDSCN method to clarify the alternated structural covariance connection patterns in mTLE and to associate them with the clinical manifestations. Moreover, we tried to illustrate the relationship between IDSCN and regional brain atrophy, which could deepen our understanding of the neuronal mechanisms of SCN changes in mTLE. Our study emphasizes the significance of hippo-thalamic-basal-cortical circuits in the pathophysiologic process of disrupted whole-brain morphological covariance networks in mTLE, and establishes a connection between global covariance network changes and regional brain atrophy.

## 2 Materials and methods

### 2.1 Participants

Our study studied 71 patients clinically diagnosed with medically refractory mTLE with hippocampal sclerosis in the Department of Neurosurgery, Xuanwu Hospital, Capital Medical University from September 1, 2019, to July 1, 2022. Each patient enrolled in the cohort was selected according to the following criteria: (1) Chinese ancestry, age between 15 and 60 years old; (2) diagnosis of mTLE with unilateral hippocampal sclerosis; and (3) having 3D T1 weighted structural MRI (sMRI) examination before surgery. Patients with clinically suspected epileptogenic lesions beside the medial temporal lobe, visible intracranial lesions (i.e., tumors and cerebrovascular diseases) or other neurological and psychiatric disorders were excluded from our cohort. Sixty-eight of 71 participants received further epilepsy resection, radiofrequency thermocoagulation or laser interstitial thermotherapy after multidisciplinary noninvasive presurgery evaluation based on clinical symptomatology, neuroimaging and neuroelectrophysiology findings. We concurrently recruited 74 healthy adults as healthy controls (HCs) in our study. Healthy controls were recruited with the following criteria: (1) Chinese ancestry, age between 18 and 30 years old; (2) no visible intracranial lesions; and (3) no other psychiatric disorders or neurological disorders.

This study was approved by the ethics committee at Xuanwu Hospital and conducted in accordance with the Declaration of Helsinki. All patients provided informed consent prior to their inclusion in the study.

### 2.2 Data acquisition

For MRI data, the sMRI images of all patients and healthy controls were collected on a 3 T GE SIGNA Premier scanner (General Electric, Fairfield Connecticut, USA) using a brain volume imaging (BRAVO) sequence. The parameters for the sMRI images were as follows: repetition time (TR) = 2,476 ms, echo time (TE) = 2.7 ms, inversion time (TI) = 0.9 s, voxel size = 1.0 mm × 1.0 mm × 1.0 mm, field of view (FOV) = 2.56*0.8594 mm, and flip angle = 8°.

For clinical data, the duration of epilepsy was defined as the time from first seizure onset to the current admission. Seizure frequency represents the average number of seizure episodes per month. Surgical outcomes of mTLE patients were assessed according to a one-year follow-up period through phone calls and outpatient visits. Patients who reached Engle class I of the Engel classification system (Engel Jr, 1993) were divided into the seizure-free (SF) group, and the remaining patients were ascribed to the seizure relapse (SR) group (Engel II-IV).

### 2.3 Image preprocessing

Voxel-based morphometry analysis was performed using the CAT12 toolbox^1^ and SPM12.^2^ First, each subject’s structural sMRI data were segmented into different tissue components, including gray matter (GM), white matter (WM), and cerebrospinal fluid (CSF). Each GM map was normalized into the Montreal Neurological Institute (MNI) space using a DARTEL algorithm and was modulated by Jacobian determinants to generate the absolute gray matter volume (GMV) (Ashburner, 2007). Finally, the normalized GM maps were smoothed using an 8-mm cubic full width at half maximum (FWHM) Gaussian kernel, resampled to 1.5 × 1.5 × 1.5 mm^3^. The total intracranial volume (TIV) was also calculated for further statistical analysis.

### 2.4 IDSCN construction and network statics

We adopted a recently published approach named the IDSCN (Liu et al., 2021) to measure individual-specific morphological covariance. First, we parcellated the brain into 116 regions of interest (ROIs) based on the automated anatomical labeling (AAL) atlas (Tzourio-Mazoyer et al., 2002) and extracted the GMV of each brain region in mTLE patients and HCs. Second, a reference structural covariance network (rSCN) was reconstructed for the HC group by calculating the partial Pearson correlation coefficient (PCC) between the pairwise GMV of any two brain regions with age, sex and TIV regarded as covariates. Then, a perturbed structural covariance network (pSCN) was constructed by adding a patient k into the HC group with the same PCC method as the rSCN. The difference between each individual’s pSCN and the rSCN (△SCN = pSCN – rSCN) was regarded as a disturbance of individual patient differences with the reference network. Finally, the Z score of △SCN (termed IDSCN) was obtained based on the equation as follows:


$$z=\frac{\mathrm{\Delta SCN}}{\left(1-rSCN^{2}\right)/\left(n-1\right)}$$

The connections of individual mTLE patients’ IDSCNs were considered significantly abnormal from the HC group rSCNs if the statistics survived after Bonferroni correction (*p* < 0.05, 116 × 115/2 = 6,670 connections).

Additionally, aiming to validate the generalizability of the results, we constructed each mTLE patients’ validated IDSCN (vIDSCN) based on a larger Brainnetom Atlas (BNA)^3^ with 246 regions of interests.

### 2.5 NBS statistics

To clarify the overall alternation pattern of mTLE, we first left–right flipped the IDSCN network of right-sided mTLE patients considering the laterality difference in mTLE. Thus, the left side of the IDSCN represents the ipsilesional side of epileptic foci, and the right side correspondingly represents the contralesional side. Furthermore, a one-sample sign permutation test was applied to the IDSCN of all mTLE patients to identify abnormal connections that deviated from norms utilizing a network-based statistics (NBS) tool (Zalesky et al., 2010) (5,000 permutations, clusterwise FWE corrected *p* < 0.05). We also identified the abnormal connections at the subject-level directly by the *Z*-distribution of the IDSCNs (Liu et al., 2021). The connections of each patient’s IDSCNs were considered significantly abnormal from the HC group rSCNs if the statistics survived after Bonferroni correction (*p* < 0.05, corrected by 116 × 115/2 = 6,670 comparisons).

Then, the number of abnormal connections (NBS statistics) and the mean number of abnormal connections per patient of each brain region (subject-level statistics) in the cohort were counted, and the top 6 brain regions were selected for further analysis.

In addition, one-sample *t*-test was applied to the vIDSCN of mTLE patients aiming to validate abnormal connections using a Graph Theoretical Network Analysis (GRETNA) toolbox (Wang et al., 2015) (1,000 permutations, *p* < 0.05, NBS correction).

### 2.6 Association between abnormal IDSCN and clinical features

A Wilcoxon rank-sum test was applied to compare the IDSCN differences between patients with SF and SR (*p* < 0.05). In addition, A Spearman correlation was applied to the correlation test between IDSCN features and continuous variables such as duration and frequency of seizure onset (*p* < 0.05).

### 2.7 Association between IDSCN and regional GMV change

A two-sample sample t test was used to determine which brain subregions suffered significant GMV loss in mTLE patients (*p* < 0.05, false discovery rate [FDR] correction). We further calculated the ROI-wise GMV atrophy rate in mTLE patients relative to the mean value among HCs. Spearman’s correlation was used to measure the correlation between regional atrophy rates across ROIs and group-wise mean values of the IDSCNs between the candidate seeds and all other ROIs (*p* < 0.05, Bonferroni correction).

### 2.8 Demographic statistics

Demographic analysis was performed with the Statistical Package for the Social Sciences version 26.0 (SPSS). A two-sample sample t test (normal distribution) or Mann–Whitney U test (nonnormal distribution) was used to compare differences in continuous variables between the mTLE and HC groups. The chi-square test was additionally used to compare differences in categorical variables such as sex and clinical status. Findings in those correlation analyses will be reported as significant if they reach a *p* < 0.05.

## 3 Results

### 3.1 Clinical characteristics

Our study included 71 patients (37 males and 34 females, aged 30.44 ± 9.39 years) diagnosed with mTLE with hippocampal sclerosis. Thirty-six patients were diagnosed with left hippocampal sclerosis, while 35 were diagnosed with right hippocampal sclerosis. Seventy-four healthy adults (34 males and 40 females, aged 24.82 ± 3.10 years) were included as healthy controls in our study. There was a significant difference in age between the patient group and the healthy control group (*t* = 4.792, *p* < 0.001), and there was no significant difference in sex (*χ*^2^ = 0.458, *p* = 0.508) or TIV (*t* = −1.690, *p* = 0.093). To remove the potential effects of age, sex and TIV on IDSCN quantification, they were considered nuisance covariates during IDSCN calculation. Table 1 depicts details of the clinical characteristics of mTLE patients and healthy controls.

**Table 1:** Demographic information of enrolled participants.

	Number of Subjects	Age (years)	Gender (M/F)	TIV (cm^3^)	Surgical treatments (ATL/RF-TC/LITT/NS)	Surgical Outcome at 1 year Follow-up (SF/SR/LF)	Duration of Epilepsy (years)	Seizure Frequency (per month)
mTLE	71	30.44 ± 9.39	37/34	1447.91 ± 173.16	48/16/4/3	30/13/28	13.08 ± 10.56	11.06 ± 24.03
HC	74	24.82 ± 3.10	34/40	1494.15 ± 156.20	–	–	–	–
Statistics	–	*t* = 4.792	*χ*^2^ = 0.458	*t* = −1.690	–	–	–	–
*p* values	–	*p* < 0.001	*p* = 0.508	*p* = 0.093	–	–	–	–

mTLE, mesial temporal lobe epilepsy; HC, healthy controls; TIV, total intracranial volume; ATL, anterior temporal lobectomy; RF-TC, radiofrequency thermocoagulation; LITT, laser interstitial thermotherapy; NS, non-surgery; SF, seizure-free (Engle I); SR, seizure relapse; LF, loss to follow-up.

### 3.2 Aberrant IDSCN pattern in mTLE patients

**Figure 1:**
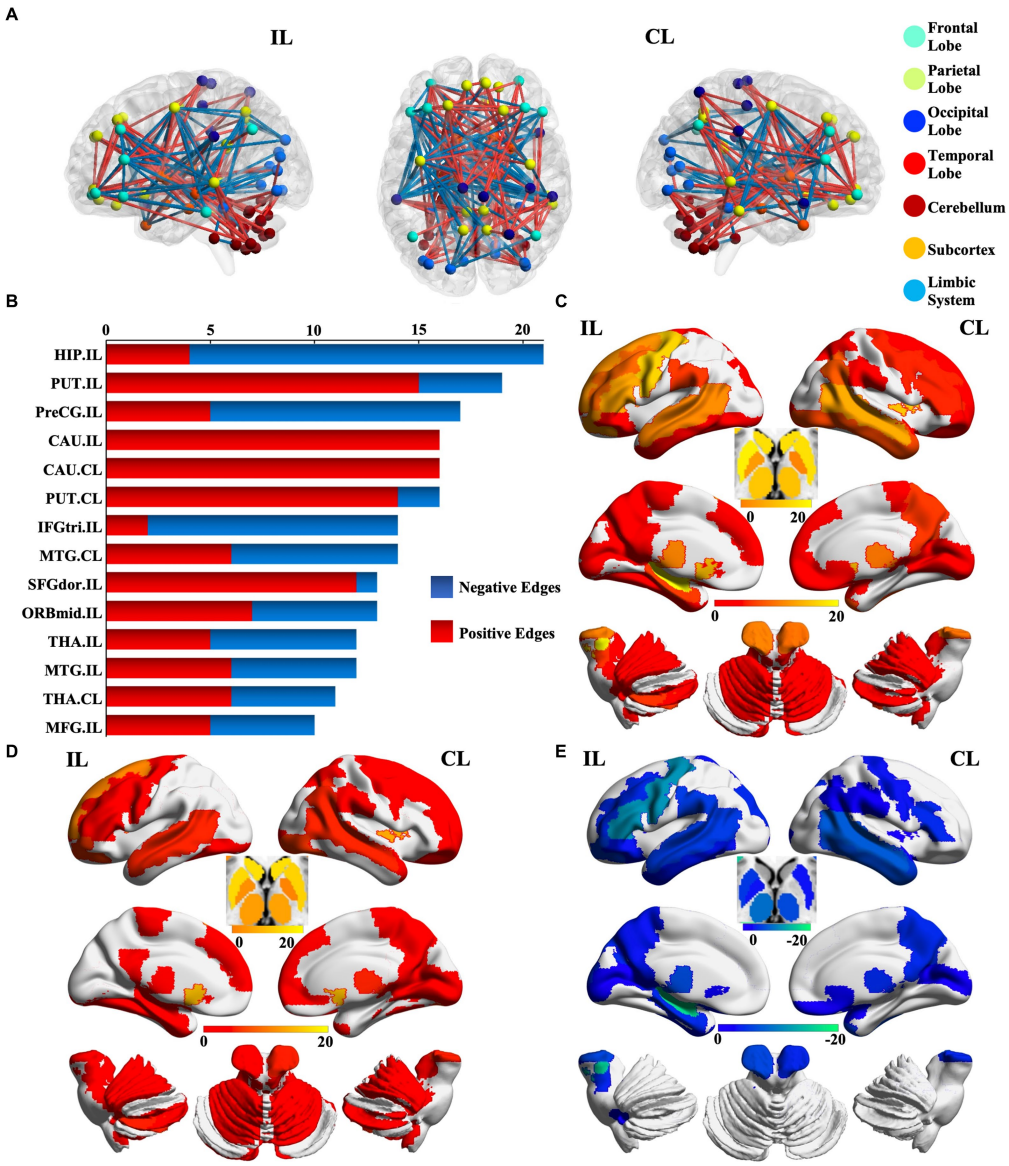
Aberrant IDSCN network in patients with mTLE. **(A)** represent the global aberrant IDSCN network in patients with mTLE. **(B)** represent the top 14 brain regions with the most significant IDSCN abnormalities. **(C)** represent the number of total aberrant connections of brain regions with significant IDSCN abnormalities. **(D,E)** represent the number of positive and negative aberrant connections of brain regions with significant IDSCN abnormalities, respectively. The full names of the brain regions are shown in Supplementary Table 1. IL, ipsilesional; CL, contralesional.

**Figure 2:**
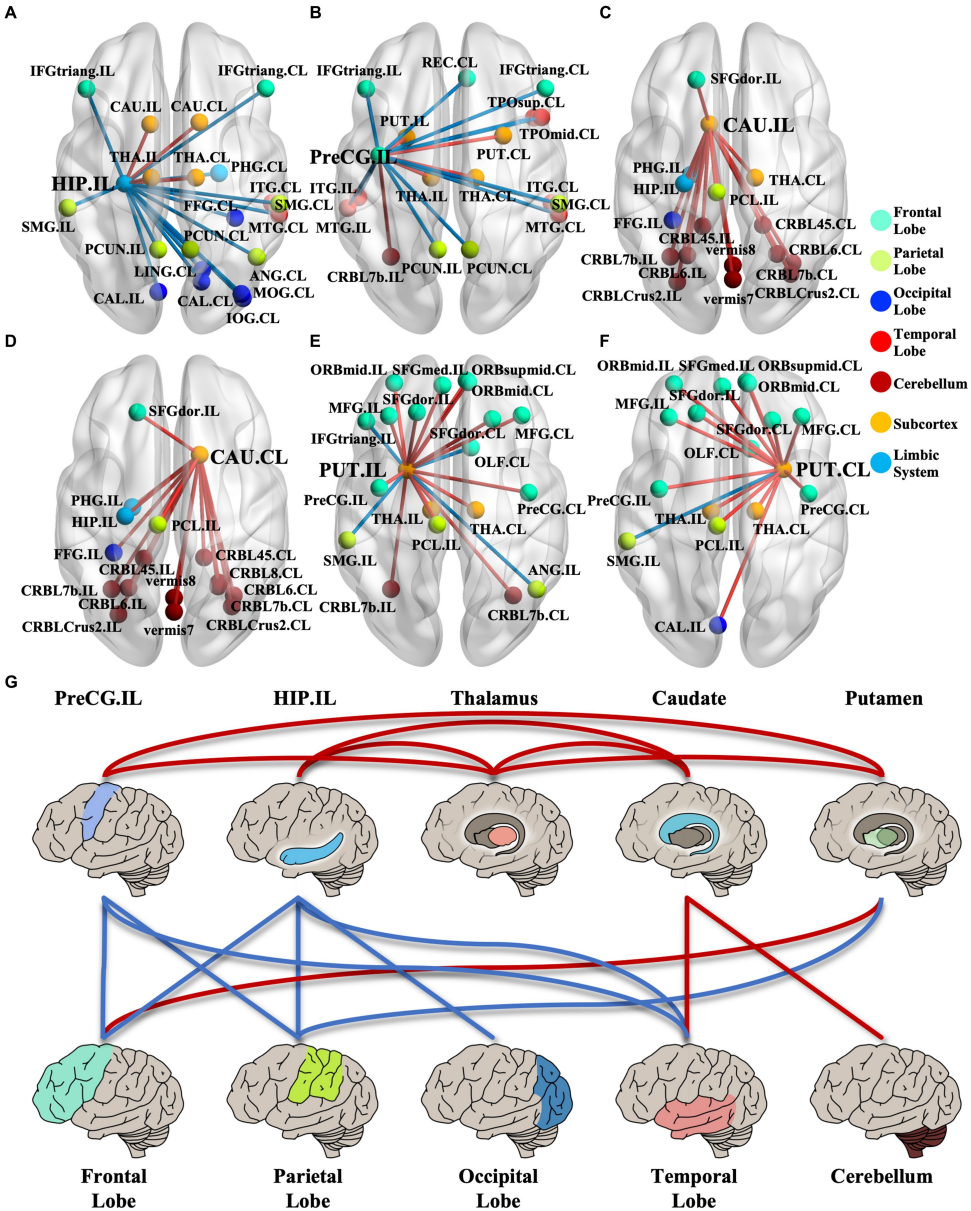
IDSCN abnormality patterns of cortical–subcortical network. **(A–F)** represents IDSCN abnormality patterns of ipsilesional hippocampus, ipsilesional precentral gyrus, ipsilesional caudate, contralesional caudate, ipsilesional putamen and contralesional putamen, respectively. **(G)** summarizes the major IDSCN alterations of cortical–subcortical network. The full names of the brain regions are shown in Supplementary Table 1. IL, ipsilesional; CL, contralesional.

We identified 214 abnormal connections among a total of 6,670 connections in mTLE patients using NBS (5,000 permutations, FWE correlation, *p* < 0.05) (Figure 1A). A total of 125 connections exhibited increased structural covariance, while 89 connections showed decreased structural covariance. Then, we measured the IDSCN abnormality of each brain region by calculating the number of abnormal connections. The top 6 brain regions with the most significant abnormalities (Figure 1B) included the ipsilesional hippocampus (4 Pos, 15 Neg), ipsilesional precentral gyrus (5 Pos, 12 Neg), bilateral caudate (ipsilesional 16 Pos; contralesional 16 Pos) and bilateral putamen (ipsilesional 15 Pos, 4 Neg; contralesional 15 Pos, 1 Neg) (Figures 1C–E). The ipsilesional hippocampus demonstrated a major decreased covariation with the bilateral frontal lobe, parietal lobe, occipital lobe, and contralesional temporal lobe and increased covariation with the bilateral thalamus and caudate (Figure 2A). The ipsilesional precentral gyrus major showed decreased covariation with the bilateral frontal lobe, temporal lobe, and parietal lobe and increased covariation with the bilateral thalamus and putamen (Figure 2B). A similar IDSCN abnormality pattern was found in the bilateral caudate with increased covariation with ipsilesional mesial temporal lobe regions and bilateral cerebellum. The ipsilesional caudate additionally depicted increased structural covariance with the contralesional thalamus (Figures 2C,D). The IDSCN abnormalities of the bilateral putamen primarily manifested as increased covariation with the bilateral thalamus and bilateral frontal lobe, including the bilateral precentral gyrus (Figures 2E,F). Figure 2G summarizes the major IDSCN alterations between the hippocampus, precentral, thalamus, caudate, and putamen.

We further evaluated the mean number of affected connections per patient of each brain region at the individual level. The ipsilesional hippocampus (10.028 ± 13.241), parahippocampal gyrus (4.944 ± 9.057), amygdala (4.883 ± 12.998), precentral gyrus (2.859 ± 6.314), and putamen (2.803 ± 6.366) exhibited more frequent abnormal connections, replicating the results by NBS analysis (Supplementary Figures 1A–D).

Similar IDSCN connection abnormalities were found in vIDSCN of mTLE patients. The top 20 brain regions with the most significant abnormalities in vIDSCN were matched to AAL atlas (Supplementary Figures 2A,B) included the ipsilesional thalamus (231 Pos, 219 Neg), bilateral putamen (ipsilesional 203 Pos, 121 Neg; contralesional 106 Pos, 66 Neg), ipsilesional precentral gyrus (177 Pos, 139 Neg), ipsilesional hippocampus (73 Pos, 158 Neg) (Supplementary Figure 2E). Moreover, we calculated the mean number of affected connections per patient of each brain region in vIDSCN at the individual level. The ipsilesional hippocampus (34.549 ± 25.581), ipsilesional thalamus (23.972 ± 13.306), bilateral amygdala (ipsilesional 22.056 ± 26.595; contralesional 8.887 ± 12.529), ipsilesional putamen (10.662 ± 14.541) showed more frequent abnormal connections (Supplementary Figures 2C,D,F). The result of vIDSCN based on BNA246 atlas replicated the AAL results in both group and individual level, ensures the generalizability of our result.

### 3.3 Clinical relevance of the IDSCN

**Figure 3:**
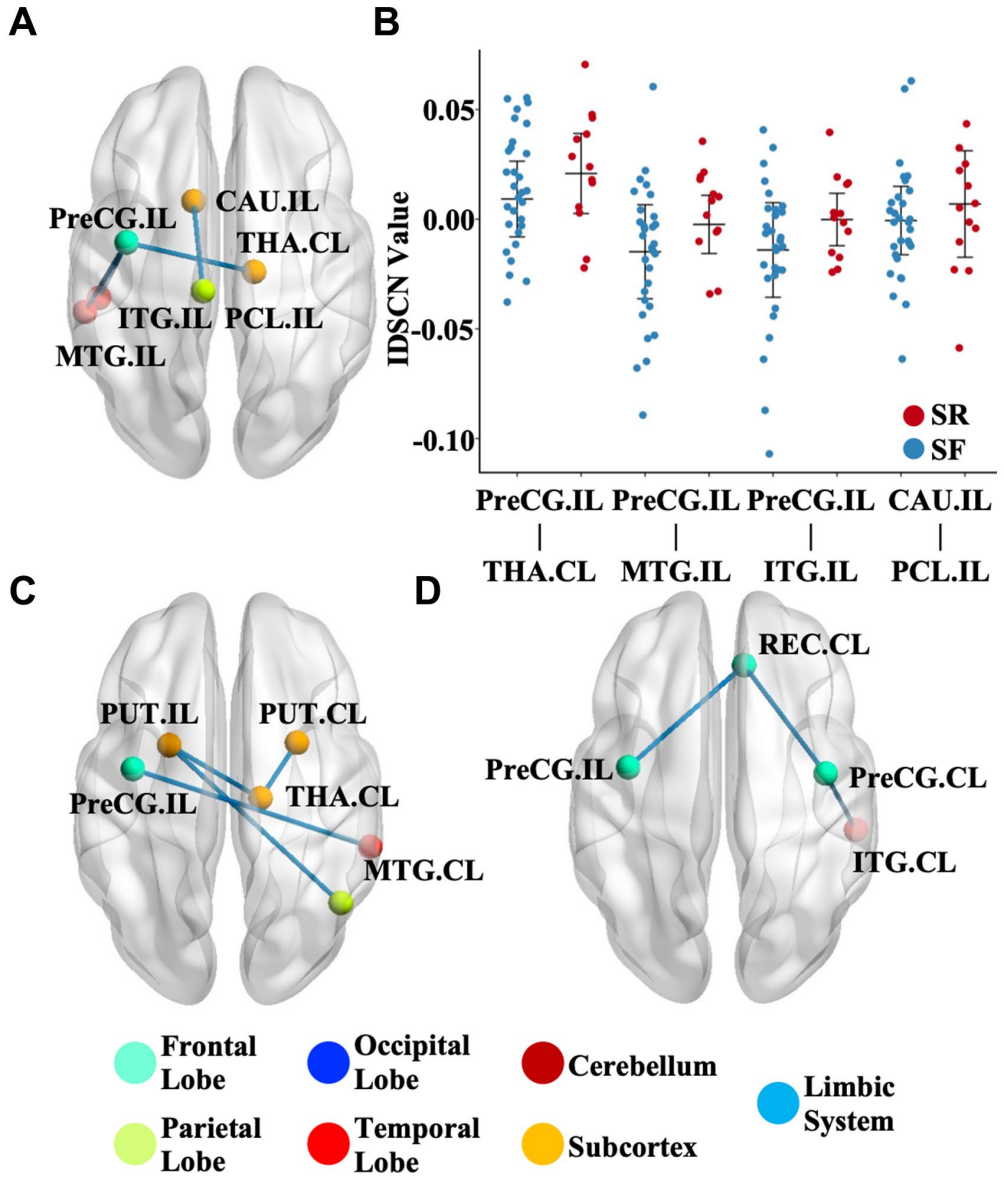
Clinical relevance of the IDSCN. **(A)** represents the differential IDSCN network between postoperatively seizure-free and seizure relapse patients. **(B)** represents the distribution of IDSCN value in postoperatively seizure-free and seizure relapse patients. **(C,D)** represents IDSCN network significantly correlated with seizure duration and seizure frequency, respectively. The full names of the brain regions are shown in Supplementary Table 1. IL, ipsilesional; CL, contralesional; SF, seizure-free; SR, seizure relapse.

We further investigated the association between the IDSCN of the six most severely involved regions (ipsilesional hippocampus, ipsilesional precentral gyrus, bilateral caudate and bilateral putamen) and clinical measures. We found that patients with SR (Engel II-IV) demonstrated significantly higher IDSCN between the ipsilesional precentral gyrus and ipsilesional middle temporal gyrus, ipsilesional inferior temporal gyrus and contralesional thalamus, and between ipsilesional caudate and ipsilesional paracentral lobule than patients with SF (Engel I) (*p* < 0.05) (Figures 3A,B). In addition, IDSCN connections between the bilateral putamen and contralesional thalamus were correlated with the duration of epilepsy (*p* < 0.05) (Figure 3C). Structural covariant alternation between the precentral gyrus and rectus gyri was found to be significantly correlated with seizure frequency (*p* < 0.05) (Figure 3D). The above results did not survive FDR correction.

### 3.4 Regional GMV changes and association with the IDSCN in mTLE

**Figure 4:**
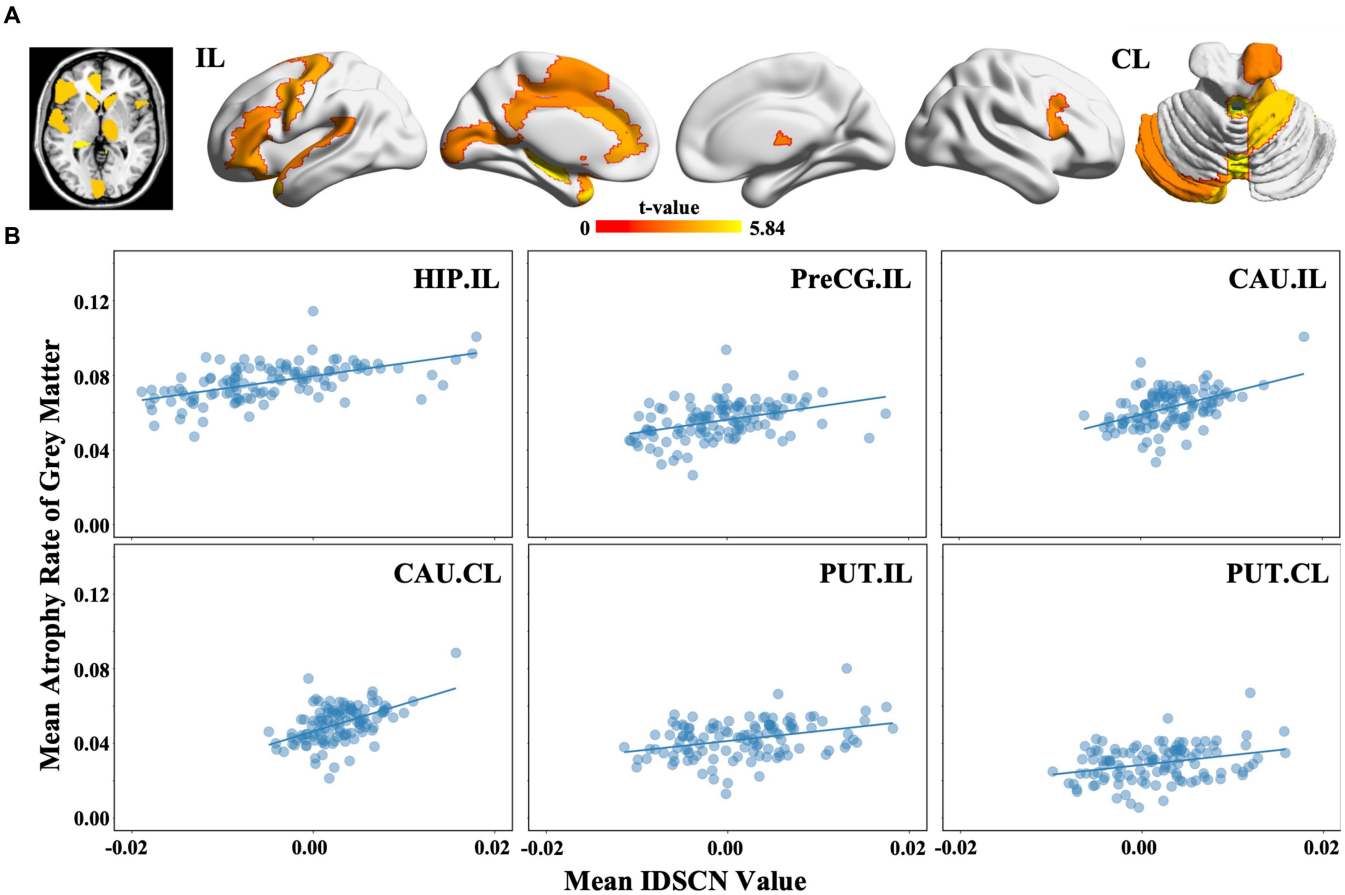
Regional gray matter volume changes and association with the IDSCN in mTLE. **(A)** represents brain regions with significant gray matter volume decreasing in mTLE patients; **(B)** represents the association between mean IDSCN value and mean trophy rate of gray matter of ipsilesional hippocampus, ipsilesional precentral gyrus, ipsilesional caudate, contralesional caudate, ipsilesional putamen and contralesional putamen with other brain areas. The full names of the brain regions are shown in Supplementary Table 1. IL, ipsilesional; CL, contralesional.

Among the 116 brain regions defined by the AAL atlas, we found that 27 subregions suffered greater GMV reduction in mTLE patients than in HCs (p < 0.05, FDR correction), including the ipsilesional hippocampus (*t* = 5.838, FDRp <0.05), ipsilesional precentral gyrus (*t* = 4.119, FDRp <0.05) and bilateral caudate (ipsilesional: *t* = 3.323, FDRp <0.05; contralesional: *t* = 3.417, FDRp <0.05) (Figure 4A). Moreover, we found that the regional atrophy rates of mTLE were spositively correlated with the mean IDSCNs of the ipsilesional hippocampus (Spearman’s correlation, *r* = 0.578, *p* < 0.001), ipsilesional precentral gyrus (Spearman’s correlation, *r* = 0.444, *p* < 0.001), bilateral caudate (Spearman’s correlation, ipsilesional *r* = 0.455, *p* < 0.001; contralesional *r* = 0.463, *p* < 0.001) and putamen (Spearman’s correlation, ipsilesional *r* = 0.326, *p* < 0.001; contralesional *r* = 0.279, *p* = 0.002) with other brain areas (Figure 4B), suggesting a close association between the severity of regional gray matter loss of these candidate regions and their structural covariance network disruptions.

## 4 Discussion

Our study aimed to demonstrate IDSCN abnormalities in mTLE patients. First, we found that mTLE patients showed characterized IDSCN disruption patterns in the ipsilesional hippocampus, ipsilesional precentral gyrus, and bilateral caudate and putamen in AAL atlas, majorly validated by a larger scale BNA brain template. In addition, the IDSCN aberrance of the six brain regions demonstrated a significant positive correlation with the regional brain atrophy rates. Finally, the abnormal IDSCN connected to the precentral gyrus and basal ganglia nucleus was weakly associated with the duration of epilepsy and postsurgery outcomes. Our findings first elucidated the IDSCN abnormalities in patients with mTLE and associated the network changes with regional brain atrophy.

Hippocampal sclerosis is recognized as a common histopathological cause of mTLE (Blumcke et al., 2013). Our study similarly found that the ipsilesional hippocampus showed the most significant structural covariance abnormalities in both AAL IDSCN and BNA IDSCN, as expected. Hippocampal sclerosis can cause extensive SCN changes. Supporting group-level SCN studies have previously illustrated hippocampal-centered network alterations in mTLE (Zhang et al., 2017). As confirmed by our research, IDSCN abnormalities of the ipsilesional hippocampus mark a vital feature of mTLE and may potentially contribute to the diagnosis of mTLE. Our study further illustrates the aberrant IDSCN connection patterns of decreased covariation with subcortical nuclei and increased covariation with cortical regions in mTLE.

Structural alternation in the precentral gyrus, the location of the primary motor center, is regarded as the effect of the synchronous electrical activity of epilepsy and is associated with seizure frequency and the duration of epilepsy in mTLE (Kassubek et al., 2014; Whelan et al., 2018). Here, we similarly found significant covariation abnormalities in the precentral gyrus. Furthermore, altered structural covariance network connections with the precentral gyrus were found to be relevant to seizure frequency and the duration of epilepsy in our cohort.

Furthermore, our study discovered morphological covariance abnormalities in the basal ganglia regions. Subcortical regions play a vital role in regulating behavior and cognition through extensive connections with cortical structures (D'Ostilio et al., 2012; Heller et al., 2016). Recently, functional (He et al., 2020) and structural (Tung et al., 2021) studies have concordantly demonstrated cortico-subcortical network alterations in temporal lobe epilepsy. The basal ganglia have been emphasized to play a significant role with multimodal effects, including sense, motor and cognition, in epilepsy patients based on an intracranial electrophysiology study (Qi et al., 2022). In our cohort, we found increased structural covariance between the basal ganglia and thalamus and extensive frontal and cerebellar regions. Synchronous discharge in epilepsy is considered associated with structural covariance alterations (Alexander-Bloch et al., 2013), marking the importance of the basal ganglia as a hub of the cortico-subcortical network in seizure generalization.

In our study, the thalamus was not in the top 6 most involved regions with disrupted IDSCNs. However, we found increased IDSCN values between the thalamus and all 6 top-affected brain regions. Meanwhile, thalamus exhibited more significant structural covariance abnormalities in vIDSCN. Indicating that the importance of the thalamus is more prominent in a more detailed brain atlas. The increased covariation corresponds to a tight anatomical coupling between the thalamus and mesial temporal lobe structures and the global cortex (Behrens et al., 2003). Both thalamus-centered structural (Keller et al., 2014) and functional (He et al., 2015) network alterations appeared in mTLE patients. Multiple pieces of evidence indicate that the thalamus is involved in temporal lobe epilepsy as a regulatory center of the hippocampal-thalamic-cortical pathway (Natsume et al., 2003; Perani et al., 2018; Chen et al., 2019), reflecting that the thalamus plays a specific role in mTLE by regulating hippocampal, basal ganglia, and cortical region function. Thus, our findings highlights the role of hippo-thalamic-basal-cortical circuits in the pathophysiologic process of disrupted whole-brain morphological covariance networks in mTLE. We hypothesized that the epileptogenic zone of the hippocampus damages extensive cortical structures mediated by the thalamus and basal ganglia through the hippo-thalamic-basal-cortical loop, which should be validated in the future.

The IDSCN was initially applied to explore neurological and psychiatric diseases (Liu et al., 2021; Han et al., 2022), but its neurophysiological mechanisms, especially the association with gray matter alterations, are still indeterminant. A growing number of studies have indicated that mTLE is a global brain network disorder involving brain areas beyond those housing focal temporal lesions (Keller et al., 2014; de Campos et al., 2016). The worldwide multicenter ENIGMA study observed brain atrophy in a wide range of cortical and subcortical regions (Whelan et al., 2018). Their findings echoed our results of gray matter volume reductions in the hippocampus, precentral gyrus, basal ganglia, and thalamus, indicating extratemporal network alterations in mTLE patients. Moreover, our study revealed that IDSCN patterns are positively associated with regional GMV reduction in mTLE patients, implying that a higher IDSCN value corresponds to a higher atrophy rate between pairs of cerebral regions. The IDSCN-atrophy association may be attributed to neuron loss secondary to neurotoxicity in epileptic seizure spreading. Our study builds a bridge between brain-wide covariance network changes and regional brain atrophy for the first time, which may deepen understanding of the neurobiological basis of structural covariance network disruption after mTLE.

Several limitations should be mentioned in our study. First, this study is based on a single-center cohort. Cross-validation of our findings would be beneficial. Second, structural covariance is reportedly linked to cognitive alterations (Seidlitz et al., 2018). Due to the limitation of the lack of cognition evaluation in our dataset, the relationship between the IDSCN and cognitive change in mTLE still needs further exploration. Third, the longitudinal process of mTLE disease progression and postoperative connection reorganization remains a limitation of our cross-sectional study.

## 5 Conclusion

In conclusion, our study applied the IDSCN to reveal the structural covariance network disruption patterns and associated them with regional gray matter atrophy of patients with medial temporal lobe epilepsy for the first time. Our findings highlights the important role of hippo-thalamic-basal-cortical circuits in the pathophysiologic process of disrupted whole-brain morphological covariance networks in mTLE, and builds a bridge between brain-wide covariance network changes and regional brain atrophy.

## Data availability statement

The original contributions presented in the study are included in the article/Supplementary material, further inquiries can be directed to the corresponding author.

## Ethics statement

The studies involving humans were approved by the Ethics Committee at Xuanwu Hospital. The studies were conducted in accordance with the local legislation and institutional requirements. Written informed consent for participation in this study was provided by the participants’ legal guardians/next of kin.

## Author contributions

YH: Data curation, Formal analysis, Methodology, Software, Validation, Visualization, Writing – original draft, Writing – review & editing. NW: Data curation, Formal analysis, Software, Writing – review & editing. WL: Visualization, Writing – review & editing. TF: Data curation, Resources, Writing – review & editing. HZ: Data curation, Resources, Writing – review & editing. XF: Data curation, Resources, Writing – review & editing. SC: Data curation, Resources, Writing – review & editing. YW: Data curation, Resources, Writing – review & editing. YS: Conceptualization, Data curation, Funding acquisition, Project administration, Resources, Supervision, Validation, Writing – review & editing. PW: Conceptualization, Data curation, Funding acquisition, Project administration, Resources, Supervision, Validation, Writing – review & editing. GZ: Conceptualization, Data curation, Funding acquisition, Project administration, Resources, Supervision, Validation, Writing – review & editing.

## Footnotes

^1^ https://dbm.neuro.uni-jena.de/cat12

^2^ https://www.fil.ion.ucl.ac.uk/spm/software/spm12/

^3^ http://atlas.brainnetome.org/index.html
