# Supplementary material for: Aberrant individual structure covariance network in patients with mesial temporal lobe epilepsy
Source: Front Neurosci. 2024 May 9;18:1381385. doi: 10.3389/fnins.2024.1381385 (PMC11112066; doi:10.3389/fnins.2024.1381385)
Supplement: Supplementary file 1 [file Data_Sheet_1.docx]

**Supplemental Table 1: Full names of brain regions in AAL atlas.**

| Full names of brain regions | Abbreviations |
| --- | --- |
| Precentral gyrus | PreCG |
| Superior frontal gyrus, dorsolateral | SFGdor |
| Superior frontal gyrus, orbital part | ORBsup |
| Middle frontal gyrus | MFG |
| Middle frontal gyrus, orbital part | ORBmid |
| Inferior frontal gyrus, opercular part | IFGoperc |
| Inferior frontal gyrus, triangular part | IFGtriang |
| Inferior frontal gyrus, orbital part | ORBinf |
| Rolandic operculum | ROL |
| Supplementary motor area | SMA |
| Olfactory cortex | OLF |
| Superior frontal gyrus, medial | SFGmed |
| Superior frontal gyrus, medial orbital | ORBsupmed |
| Gyrus rectus | REC |
| Insula | INS |
| Anterior cingulate and paracingulate gyri | ACG |
| Median cingulate and paracingulate gyri | DCG |
| Posterior cingulate gyrus | PCG |
| Hippocampus | HIP |
| Parahippocampal gyrus | PHG |
| Amygdala | AMYG |
| Calcarine fissure and surrounding cortex | CAL |
| Cuneus | CUN |
| Lingual gyrus | LING |
| Superior occipital gyrus | SOG |
| Middle occipital gyrus | MOG |
| Inferior occipital gyrus | IOG |
| Fusiform gyrus | FFG |
| Postcentral gyrus | PoCG |
| Superior parietal gyrus | SPG |
| Inferior parietal, but supramarginal and angular gyri | IPL |
| Supramarginal gyrus | SMG |
| Angular gyrus | ANG |
| Precuneus | PCUN |
| Paracentral lobule | PCL |
| Caudate nucleus | CAU |
| Lenticular nucleus, putamen | PUT |
| Lenticular nucleus, pallidum | PAL |
| Thalamus | THA |
| Heschl gyrus | HES |
| Superior temporal gyrus | STG |
| Temporal pole: superior temporal gyrus | TPOsup |
| Middle temporal gyrus | MTG |
| Temporal pole: middle temporal gyrus | TPOmid |
| Inferior temporal gyrus | ITG |

**Supplemental Table 2: Full names of brain regions in BNA246 atlas.**

| **Gyrus** | **Abbreviation** | **Anatomical and modified Cyto-architectonic descriptions** |
| --- | --- | --- |
| SFG, Superior Frontal Gyrus | SFG_IL(CL)_7_1 | *A8m, medial area 8* |
|  | SFG_IL(CL)_7_2 | *A8dl, dorsolateral area 8* |
|  | SFG_IL(CL)_7_3 | *A9l, lateral area 9* |
|  | SFG_IL(CL)_7_4 | *A6dl, dorsolateral area 6* |
|  | SFG_IL(CL)_7_5 | *A6m, medial area 6* |
|  | SFG_IL(CL)_7_6 | *A9m,medial area 9* |
|  | SFG_IL(CL)_7_7 | *A10m, medial area 10* |
| MFG, Middle Frontal Gyrus | MFG_IL(CL)_7_1 | *A9/46d, dorsal area 9/46* |
|  | MFG_IL(CL)_7_2 | *IFJ, inferior frontal junction* |
|  | MFG_IL(CL)_7_3 | *A46, area 46* |
|  | MFG_IL(CL)_7_4 | *A9/46v, ventral area 9/46* |
|  | MFG_IL(CL)_7_5 | *A8vl, ventrolateral area 8* |
|  | MFG_IL(CL)_7_6 | *A6vl, ventrolateral area 6* |
|  | MFG_IL(CL)_7_7 | *A10l, lateral area10* |
| IFG, Inferior Frontal Gyrus | IFG_IL(CL)_6_1 | *A44d,dorsal area 44* |
|  | IFG_IL(CL)_6_2 | *IFS, inferior frontal sulcus* |
|  | IFG_IL(CL)_6_3 | *A45c, caudal area 45* |
|  | IFG_IL(CL)_6_4 | *A45r, rostral area 45* |
|  | IFG_IL(CL)_6_5 | *A44op, opercular area 44* |
|  | IFG_IL(CL)_6_6 | *A44v, ventral area 44* |
| OrG, Orbital Gyrus | OrG_IL(CL)_6_1 | *A14m, medial area 14* |
|  | OrG_IL(CL)_6_2 | *A12/47o, orbital area 12/47* |
|  | OrG_IL(CL)_6_3 | *A11l, lateral area 11* |
|  | OrG_IL(CL)_6_4 | *A11m, medial area 11* |
|  | OrG_IL(CL)_6_5 | *A13, area 13* |
|  | OrG_IL(CL)_6_6 | *A12/47l, lateral area 12/47* |
| PrG, Precentral Gyrus | PrG_IL(CL)_6_1 | *A4hf, area 4(head and face region)* |
|  | PrG_IL(CL)_6_2 | *A6cdl, caudal dorsolateral area 6* |
|  | PrG_IL(CL)_6_3 | *A4ul, area 4(upper limb region)* |
|  | PrG_IL(CL)_6_4 | *A4t, area 4(trunk region)* |
|  | PrG_IL(CL)_6_5 | *A4tl, area 4(tongue and larynx region)* |
|  | PrG_IL(CL)_6_6 | *A6cvl, caudal ventrolateral area 6* |
| PCL, Paracentral Lobule | PCL_IL(CL)_2_1 | *A1/2/3ll, area1/2/3 (lower limb region)* |
|  | PCL_IL(CL)_2_2 | *A4ll, area 4, (lower limb region)* |
| STG, Superior Temporal Gyrus | STG_IL(CL)_6_1 | *A38m, medial area 38* |
|  | STG_IL(CL)_6_2 | *A41/42, area 41/42* |
|  | STG_IL(CL)_6_3 | *TE1.0 and TE1.2* |
|  | STG_IL(CL)_6_4 | *A22c, caudal area 22* |
|  | STG_IL(CL)_6_5 | *A38l, lateral area 38* |
|  | STG_IL(CL)_6_6 | *A22r, rostral area 22* |
| MTG, Middle Temporal Gyrus | MTG_IL(CL)_4_1 | *A21c, caudal area 21* |
|  | MTG_IL(CL)_4_2 | *A21r, rostral area 21* |
|  | MTG_IL(CL)_4_3 | *A37dl, dorsolateral area37* |
|  | MTG_IL(CL)_4_4 | *aSTS, anterior superior temporal sulcus* |
| ITG, Inferior Temporal Gyrus | ITG_IL(CL)_7_1 | *A20iv, intermediate ventral area 20* |
|  | ITG_IL(CL)_7_2 | *A37elv, extreme lateroventral area37* |
|  | ITG_IL(CL)_7_3 | *A20r, rostral area 20* |
|  | ITG_IL(CL)_7_4 | *A20il, intermediate lateral area 20* |
|  | ITG_IL(CL)_7_5 | *A37vl, ventrolateral area 37* |
|  | ITG_IL(CL)_7_6 | *A20cl, caudolateral of area 20* |
|  | ITG_IL(CL)_7_7 | *A20cv, caudoventral of area 20* |
| FuG, Fusiform Gyrus | FuG_IL(CL)_3_1 | *A20rv, rostroventral area 20* |
|  | FuG_IL(CL)_3_2 | *A37mv, medioventral area37* |
|  | FuG_IL(CL)_3_3 | *A37lv, lateroventral area37* |
| PhG, Parahippocampal Gyrus | PhG_IL(CL)_6_1 | *A35/36r, rostral area 35/36* |
|  | PhG_IL(CL)_6_2 | *A35/36c, caudal area 35/36* |
|  | PhG_IL(CL)_6_3 | *TL, area TL (lateral PPHC, posterior parahippocampal gyrus)* |
|  | PhG_IL(CL)_6_4 | *A28/34, area 28/34 (EC, entorhinal cortex)* |
|  | PhG_IL(CL)_6_5 | *TI, area TI(temporal agranular insular cortex)* |
|  | PhG_IL(CL)_6_6 | *TH, area TH (medial PPHC)* |
| pSTS, posterior Superior Temporal Sulcus | pSTS_IL(CL)_2_1 | *rpSTS, rostroposterior superior temporal sulcus* |
|  | pSTS_IL(CL)_2_2 | *cpSTS, caudoposterior superior temporal sulcus* |
| SPL, Superior Parietal Lobule | SPL_IL(CL)_5_1 | *A7r, rostral area 7* |
|  | SPL_IL(CL)_5_2 | *A7c, caudal area 7* |
|  | SPL_IL(CL)_5_3 | *A5l, lateral area 5* |
|  | SPL_IL(CL)_5_4 | *A7pc, postcentral area 7* |
|  | SPL_IL(CL)_5_5 | *A7ip, intraparietal area 7(hIP3)* |
| IPL, Inferior Parietal Lobule | IPL_IL(CL)_6_1 | *A39c, caudal area 39(PGp)* |
|  | IPL_IL(CL)_6_2 | *A39rd, rostrodorsal area 39(Hip3)* |
|  | IPL_IL(CL)_6_3 | *A40rd, rostrodorsal area 40(PFt)* |
|  | IPL_IL(CL)_6_4 | *A40c, caudal area 40(PFm)* |
|  | IPL_IL(CL)_6_5 | *A39rv, rostroventral area 39(PGa)* |
|  | IPL_IL(CL)_6_6 | *A40rv, rostroventral area 40(PFop)* |
| Pcun, Precuneus | PCun_IL(CL)_4_1 | *A7m, medial area 7(PEp)* |
|  | PCun_IL(CL)_4_2 | *A5m, medial area 5(PEm)* |
|  | PCun_IL(CL)_4_3 | *dmPOS, dorsomedial parietooccipital sulcus(PEr)* |
|  | PCun_IL(CL)_4_4 | *A31, area 31 (Lc1)* |
| PoG, Postcentral Gyrus | PoG_IL(CL)_4_1 | *A1/2/3ulhf, area 1/2/3(upper limb, head and face region)* |
|  | PoG_IL(CL)_4_2 | *A1/2/3tonIa, area 1/2/3(tongue and larynx region)* |
|  | PoG_IL(CL)_4_3 | *A2, area 2* |
|  | PoG_IL(CL)_4_4 | *A1/2/3tru, area1/2/3(trunk region)* |
| INS, Insular Gyrus | INS_IL(CL)_6_1 | *G, hypergranular insula* |
|  | INS_IL(CL)_6_2 | *vIa, ventral agranular insula* |
|  | INS_IL(CL)_6_3 | *dIa, dorsal agranular insula* |
|  | INS_IL(CL)_6_4 | *vId/vIg, ventral dysgranular and granular insula* |
|  | INS_IL(CL)_6_5 | *dIg, dorsal granular insula* |
|  | INS_IL(CL)_6_6 | *dId, dorsal dysgranular insula* |
| CG, Cingulate Gyrus | CG_IL(CL)_7_1 | *A23d, dorsal area 23* |
|  | CG_IL(CL)_7_2 | *A24rv, rostroventral area 24* |
|  | CG_IL(CL)_7_3 | *A32p, pregenual area 32* |
|  | CG_IL(CL)_7_4 | *A23v, ventral area 23* |
|  | CG_IL(CL)_7_5 | *A24cd, caudodorsal area 24* |
|  | CG_IL(CL)_7_6 | *A23c, caudal area 23* |
|  | CG_IL(CL)_7_7 | *A32sg, subgenual area 32* |
| MVOcC*,* MedioVentral Occipital Cortex | MVOcC _IL(CL)_5_1 | *cLinG, caudal lingual gyrus* |
|  | MVOcC _IL(CL)_5_2 | *rCunG, rostral cuneus gyrus* |
|  | MVOcC _IL(CL)_5_3 | *cCunG, caudal cuneus gyrus* |
|  | MVOcC _IL(CL)_5_4 | *rLinG, rostral lingual gyrus* |
|  | MVOcC _IL(CL)_5_5 | *vmPOS,ventromedial parietooccipital sulcus* |
| LOcC, lateral Occipital Cortex | LOcC_IL(CL)_4_1 | *mOccG, middle occipital gyrus* |
|  | LOcC _IL(CL)_4_2 | *V5/MT+, area V5/MT+* |
|  | LOcC _IL(CL)_4_3 | *OPC, occipital polar cortex* |
|  | LOcC_IL(CL)_4_4 | *iOccG, inferior occipital gyrus* |
|  | LOcC _IL(CL)_2_1 | *msOccG, medial superior occipital gyrus* |
|  | LOcC _IL(CL)_2_2 | *lsOccG, lateral superior occipital gyrus* |
| Amyg, Amygdala | Amyg_IL(CL)_2_1 | *mAmyg, medial amygdala* |
|  | Amyg_IL(CL)_2_2 | *lAmyg, lateral amygdala* |
| Hipp, Hippocampus | Hipp_IL(CL)_2_1 | *rHipp, rostral hippocampus* |
|  | Hipp_IL(CL)_2_2 | *cHipp, caudal hippocampus* |
| BG, Basal Ganglia | BG_IL(CL)_6_1 | *vCa, ventral caudate* |
|  | BG_IL(CL)_6_2 | *GP, globus pallidus* |
|  | BG_IL(CL)_6_3 | *NAC, nucleus accumbens* |
|  | BG_IL(CL)_6_4 | *vmPu, ventromedial putamen* |
|  | BG_IL(CL)_6_5 | *dCa, dorsal caudate* |
|  | BG_IL(CL)_6_6 | *dlPu, dorsolateral putamen* |
| Tha, Thalamus | Tha_IL(CL)_8_1 | *mPFtha, medial pre-frontal thalamus* |
|  | Tha_IL(CL)_8_2 | *mPMtha, pre-motor thalamus* |
|  | Tha_IL(CL)_8_3 | *Stha, sensory thalamus* |
|  | Tha_IL(CL)_8_4 | *rTtha, rostral temporal thalamus* |
|  | Tha_IL(CL)_8_5 | *PPtha, posterior parietal thalamus* |
|  | Tha_IL(CL)_8_6 | *Otha, occipital thalamus* |
|  | Tha_IL(CL)_8_7 | *cTtha, caudal temporal thalamus* |
|  | Tha_IL(CL)_8_8 | *lPFtha, lateral pre-frontal thalamus* |


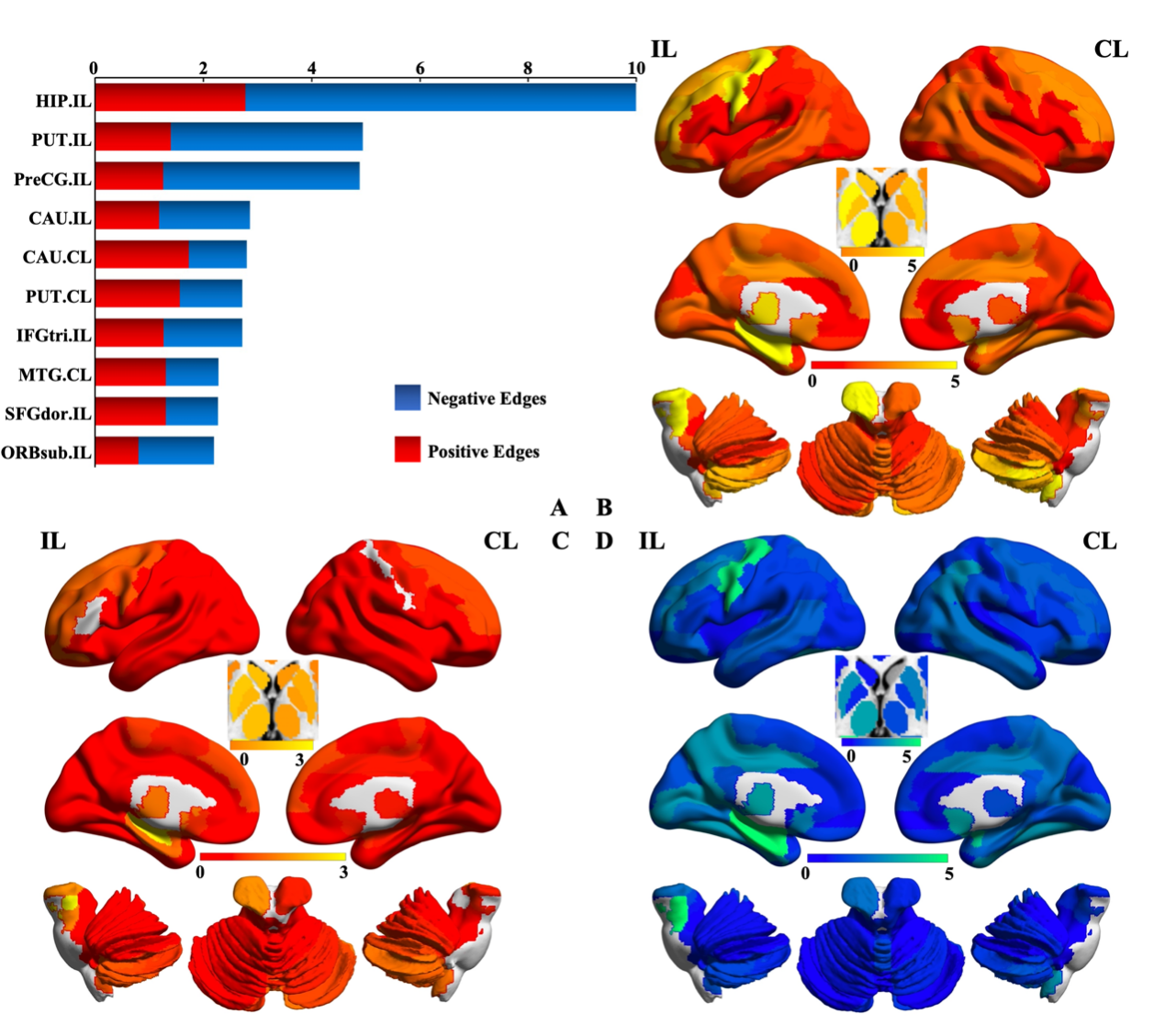


**Supplemental Figure 1: Mean number of aberrant IDSCN edges per patient of each brain region.** (A) represent the top 10 brain regions with the most aberrant IDSCN edges per patient. (B) represent the mean number of total aberrant edges per patient in separate brain regions. (C) and (D) represent the mean number of positive and negative aberrant edges per patient in separate brain regions respectively. The full names of the brain regions are shown in Supplementary Table 1.

Abbreviations: IL: ipsilesional; CL: contralesional;


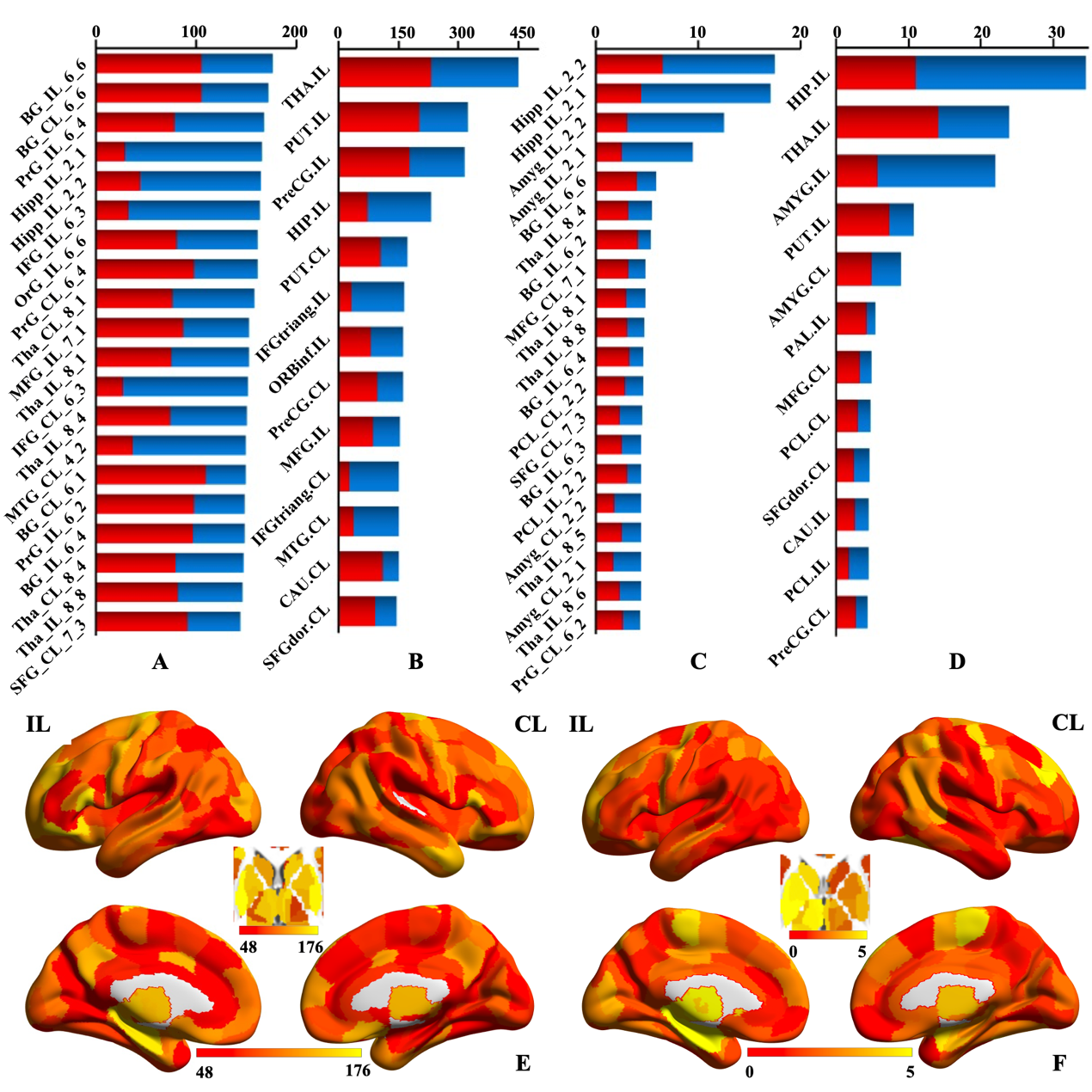


**Supplemental Figure 2: Aberrant IDSCN network and mean number of aberrant IDSCN edges per patient of each brain region (Validation IDSCN, BNA246 atlas).**

(A) represent the top 20 brain regions with the most significant IDSCN abnormalities in BNA atlas. (B) represent the AAL brain regions correspond to the top 20 brain regions with the most significant IDSCN abnormalities in BNA atlas. (C) represent the top 20 brain regions with the most aberrant IDSCN edges per patient in BNA atlas. (D) represent the AAL brain regions correspond to the top 20 brain regions with the most aberrant IDSCN edges per patient in BNA atlas. (E) represent the number of total aberrant connections of brain regions with significant IDSCN abnormalities in BNA atlas. (F) represent the mean number of total aberrant edges per patient in separate brain regions in BNA atlas.The full names of the brain regions of BNA are shown in Supplementary Table 2.

Abbreviations: IL: ipsilesional; CL: contralesional;
